# Concurrent Lead and Cadmium Exposure Among Diabetics: A Case-Control Study of Socio-Demographic and Consumption Behaviors

**DOI:** 10.3390/nu17040710

**Published:** 2025-02-17

**Authors:** Jonah Bawa Adokwe, Phisit Pouyfung, Saruda Kuraeiad, Paleeratana Wongrith, Puchong Inchai, Supabhorn Yimthiang, Soisungwan Satarug, Tanaporn Khamphaya

**Affiliations:** 1 Environmental Safety Technology and Health, School of Public Health, Walailak University, Nakhon Si Thammarat 80160, Thailand; jonahbawa.ad@mail.wu.ac.th (J.B.A.); phisit.po@wu.ac.th (P.P.); ksupapor@mail.wu.ac.th (S.Y.); 2 Department of Occupational Health and Safety, School of Public Health, Walailak University, Nakhon Si Thammarat 80160, Thailand; 3 Medical Technology, School of Allied Health Sciences, Walailak University, Nakhon Si Thammarat 80160, Thailand; saruda.ku@wu.ac.th; 4 Department of Community Public Health, School of Public Health, Walailak University, Nakhon Si Thammarat 80160, Thailand; paleeratana.wo@wu.ac.th; 5 Department of Epidemiology, Faculty of Public Health, Mahidol University, Bangkok 10400, Thailand; puchong.inc@mahidol.ac.th; 6 Kidney Disease Research Collaborative, Translational Research Institute Woolloongabba, Brisbane, QLD 4102, Australia; sj.satarug@yahoo.com.au; 7 Excellence Center for Public Health Research, Walailak University, Nakhon Si Thammarat 80160, Thailand

**Keywords:** lead, cadmium, type 2 diabetes, environmental factor

## Abstract

**Introduction/Objectives**: Type 2 diabetes (T2D) continues to pose a substantial global public health challenge. Current evidence has linked an increase in the risk of T2D to chronic exposure to the heavy metals cadmium (Cd) and lead (Pb). The present study aimed to examine whether the reported links existed in an area of southern Thailand with known Pb contamination. **Materials and Methods**: A case–control study design was used to recruit 88 diagnosed T2D cases and 90 age-, gender- and locality-matched non-diabetic controls. Blood levels of Cd and Pb were used as exposure indicators. Exposure-related risk factors and socio-demographic data were collected through questionnaires. **Results**: A significant association was found between blood Pb and T2D diagnosis, but the association between blood Cd and T2D was not statistically significant. Factors related to high Pb exposure were education, occupation, income, smoking habits, alcohol consumption, and dietary patterns, particularly the consumption of sweet and fatty foods. Participants with higher blood Pb levels had poorer glycemic control, thereby suggesting potential interference of Pb with oral hypoglycemic agents. **Conclusions**: This study confirms the connection between Pb exposure and increased risk of having T2D. Additionally, it identified socio-demographic factors, and consumption habits that contributed to such an enhanced T2D risk. The role of Cd exposure requires further studies, using urinary Cd excretion, which reflects long-term exposure conditions. These findings suggest the need to incorporate environmental and occupational exposure in diabetes care strategies. From the clinical and public health perspectives, targeted interventions should focus on reducing heavy metal exposure, improving risk awareness, and strengthening occupational safety measures to prevent disease progression.

## 1. Introduction

The global prevalence of diabetes has experienced a significant increase, rendering it a critical concern in public health. The World Health Organization’s report delineates that approximately 250 million individuals are afflicted with diabetes, and projections indicate that this figure is poised to surpass 360 million by the year 2030 [1]. The growing burden of diabetes precipitates considerable socioeconomic and healthcare ramifications [2]. Type 2 diabetes (T2D) is known to inflict extensive damage upon various organs, including the kidneys, eyes, heart, and vascular system. Consequently, this can culminate in a range of intricate health complications such as cerebrovascular accidents, myocardial infarctions, and renal failure. Established risk factors involve genetic predisposition, obesity, physical inactivity, tobacco consumption, and alcohol abuse. Nonetheless, emerging data derived from animal experimentation and epidemiological investigations imply that environmental exposure to heavy metals, which pervade our surroundings, may also contribute to T2D risk [3,4,5].

Heavy metals constitute a significant health hazard owing to their pronounced toxicity and propensity for bioaccumulation within organisms. These metals are extensively distributed throughout the environment, originating from diverse sources such as geogenic, industrial, agricultural, domestic effluents, and atmospheric emissions [6]. Human exposure to heavy metals may result from residing in high-risk zones, engaging in specific occupations (e.g., ship repair, mining, and agriculture), ingesting contaminated food and beverages, and utilizing herbal products [7,8,9]. Empirical evidence derived from both animal and epidemiological research has demonstrated that exposure to heavy metals, including lead, cadmium, mercury, and arsenic, can elevate the risk of chronic ailments such as type 2 diabetes (T2D), diabetic nephropathy, and hypertension related to diabetes [3,8,10,11,12].

Previous research has revealed a potential association between heavy metal exposure and diabetes, as demonstrated by epidemiological studies that identified a positive correlation between urinary cadmium concentrations and T2D at levels exceeding 2.4 μg/g creatinine [13]. However, despite these findings, the relationship between environmental exposure to heavy metals and diabetes incidence remains inconclusive. Inconsistencies in prior epidemiological studies may stem from variations in study design, reliance on self-reported T2D cases, and the failure to assess co-exposure to multiple metals rather than individual metal exposure.

Diabetes constitutes a principal contributor to mortality in Thailand, which harbors the seventh-largest diabetic population within the Western Pacific region [14]. A prevalence rate of 9.9% has been documented among the adult demographic [15]. Annually, diabetes is accountable for approximately 30,000 fatalities in Thailand. Concurrently, environmental lead contamination has been detected among individuals employed in lead-associated professions and children residing in proximity to fishing communities in the Nakhon Si Thammarat province [16,17]. Given these concerns, a more detailed epidemiological assessment is necessary to understand the implications of heavy metal exposure on diabetes risk in affected populations.

This study aims to bridge this gap by conducting a case–control study to investigate the correlation between lead and cadmium exposure and T2D. The objective is to identify relevant risk factors correlated with blood lead and cadmium concentrations, thereby offering guidelines for improved diabetes management and risk prediction. By employing a structured epidemiological approach, this study seeks to provide valuable insights into the interplay between environmental contaminants and diabetes prevalence, facilitating more targeted public health interventions.

## 2. Materials and Methods

This study was conducted following the Strengthening the Reporting of Observational Studies in Epidemiology (STROBE) guidelines [18]. The STROBE checklist is included as a Supplementary File (Supplementary Table S1) to ensure the completeness and transparency of the study design and reporting.

### 2.1. Aims and Research Questions

The primary objective of this study was to investigate the association between lead (Pb) and cadmium (Cd) exposure and the risk of type 2 diabetes (T2D) in individuals residing in regions with high environmental contamination while the secondary objectives were (i) to assess the sociodemographic and lifestyle factors influencing Pb and Cd exposure in diabetic and non-diabetic individuals; (ii) to determine the levels of blood Pb and Cd among study participants and evaluate their potential role as biomarkers for T2D risk, and (iii) to explore possible confounding variables affecting the relationship between heavy metal exposure and diabetes risk. In addition, the research questions consist of the following: (i) Is there a significant association between blood Pb and Cd levels and T2D risk? (ii) How do sociodemographic and lifestyle factors influence Pb and Cd exposure in the study population? (iii) Can blood Pb and Cd levels serve as predictive biomarkers for T2D in environmentally exposed populations?

### 2.2. Participants

This case–control study was designed to include 88 diabetic subjects and 90 non-diabetic control individuals. The age- and gender-matched control group was recruited using a purposive sampling method from the local health center in Pakpoon municipality. The study was conducted in a specific geographic area where the contamination of Pb and Cd is higher than in other regions. Data collection took place from January 2020 to December 2020. The study adhered to ethical standards approved by the Office of the Human Research Ethics Committee of Walailak University, with the issuance number WUEC-21-223-01. All participants were informed about the objectives, study procedures, benefits, and potential risks of the study. Subsequently, they provided written informed consent and assent for participation.

### 2.3. Sociodemographic and Exposure Data

Sociodemographic data related to lead and cadmium exposure were collected, including factors such as age, gender, marital status, education, occupation, health status, family history of diabetes mellitus, waist and hip circumference, environmental tobacco smoke exposure, alcohol consumption, and physical activity. This information was obtained using structured interview questionnaires administered by trained personnel. Additionally, body mass index (BMI) and blood pressure were measured before collecting blood and urine samples. Each participant’s blood pressure was evaluated using an electronic blood pressure monitor on their right upper arm. Trained technicians measured the participants’ weight and height while they were barefoot and dressed in light clothing. The BMI was subsequently calculated by dividing the weight in kilograms by the height in meters squared.

### 2.4. Blood and Urine Sampling

Following an overnight fast, blood and morning urine samples were simultaneously collected from each participant to measure fasting blood sugar, as well as cadmium (Cd) and lead (Pb) levels in whole blood. The fasting blood sugar was measured using an enzymatic colorimetric method with a glucose oxidase–peroxidase (GOD-POD) system, which provides accurate and reproducible results. To ensure precision in heavy metal analysis, strict protocols were followed. A 3 mL venous blood sample was drawn from each participant using sterile techniques and collected into ethylene diamine tetra-acetic acid (EDTA) tubes. Urine samples were collected in metal-free polypropylene tubes to prevent contamination. The collected blood and urine samples were immediately stored at −20 °C until analysis to maintain sample integrity. Additionally, internal quality control measures were implemented. Duplicate samples were analyzed to check for consistency, and blank samples were included to monitor contamination during processing. Certified reference materials (CRMs) were used to validate the accuracy of the blood Pb and Cd measurements. The entire process adhered to Good Laboratory Practice (GLP) standards to ensure data reliability.

### 2.5. Lead and Cadmium Analysis

Graphite furnace atomic absorption spectrometry (GBC System 5000 Graphite furnace and 902 atomic absorption spectrophotometer, PAL2000 autosampler, 902 double beam spectrophotometer, GBC Scientific Equipment, Hampshire, IL, USA) was employed for lead and cadmium analysis following the method described by Trzcinka-Ochocka et al. (2016) [19]. The limits of detection for Pb in blood were 3 µg/dL, while the limits of detection for Cd in urine and blood were 0.1 µg/L. Multi-element standards were used to calibrate Pb and Cd analysis. For quality control, the analytical accuracy and precision of these analyses were evaluated using reference urine and whole blood metal control levels 1, 2, and 3 (Lyphocheck, Bio-Rad, Hercules, CA, USA). To maintain analytical accuracy, external quality assessments for Pb and Cd detection were conducted every three years. All plastic tubes, bottles, and pipettes used in the analysis were acid-washed and rinsed with deionized water.

### 2.6. Body Composition Measurement

Anthropometric measures including height and weight were obtained using a height and weight scale machine (NAGATA MODEL BW-1122H, Tainan, Taiwan) for body mass index (BMI) calculation (kg/m^2^). BMI was divided into four groups: (i) thin (BMI < 18 kg/m^2^), (ii) normal (18 ≤ BMI < 23 kg/m^2^), (iii) overweight (23 ≤ BMI < 29 kg/m^2^), and (iv) obese (BMI ≥ 29 kg/m^2^). According to WHO protocol, waist (midpoint between the lower border of the rib cage and the iliac crest) and hip (the fullest part of the hip) circumferences were determined using measuring tape (Seca, Hamburg, Germany). Waist-to-hip ratios were then calculated of which optimal cut-off for overweight and obesity were values of 0.8 and 1.0 for females and males, respectively. Body composition analyzer model SC-330 (Tanita Health Equipment, Kowloon, Hong Kong) was employed to determine different aspects of body composition including muscle mass (kg), body fat percentage (fat mass), visceral fat rating scale, bone mass (kg), and total body water (%). For fat mass percentage, there are four categories including (1) below healthy body fat percentage, (2) healthy body fat percentage, (3) overfat (above the healthy range), and (4) obesity (high above the healthy range). The visceral rating scale range from 1 to 12 was considered a healthy level of visceral fat. For healthy total body water percentage, values between 45 and 60% for females and between 50 and 65% for males were considered. Basal metabolic rate (minimum level of energy required for body activity) was calculated based on weight, age, and muscle mass.

### 2.7. Statistical Analysis

Statistical analysis was performed using SPSS 17.0 (SPSS Inc., Chicago, IL, USA). The distribution of continuous variables was first assessed by the Shapiro–Wilk test. For normally distributed variables, the independent *t*-test was used to compare two groups. For non-normally distributed variables, the Mann–Whitney U test was used to assess the differences between two groups, while the Kruskal–Wallis test was used to compare three or more groups, followed by a post hoc test. The Chi-square test was used to determine differences in percentage. Binary logistic regression analysis was conducted to determine odds ratios (ORs) for high blood lead levels, adjusting for potential confounders. For all tests, a *p*-value of less than 0.05 indicates a statistically significant difference.

## 3. Results

### 3.1. Socio-Demographic and Anthropometric Differences in T2D and Controls

A case–control study was conducted to investigate the relationship between environ- mental lead exposure, sociodemographic factors, and type 2 diabetes (T2D). The study enrolled 178 participants, including 88 T2D cases and 90 non-T2D controls matched by age and gender. The general characteristics of the T2D and non-T2D groups are presented in Table 1. Both T2D and control groups were predominantly women (80.70% and 81.10%, respectively) with an average age of 60 years in the T2D group and 59 years in the non-T2D control group. There was no significant difference in body mass index (BMI) between the two groups. However, the average waist circumference of the T2D group (95.15 cm) was statistically higher than that of the control group (87.57 cm) with a *p*-value less than 0.001 (Table 1). Regarding sociodemographic factors, there were no significant differences between the T2D and non-control groups in terms of monthly income, smoking, alcohol consumption, sweet consumption score, fat consumption score, and salt consumption score (Table 1, *p* > 0.05). However, there were significant differences in marital status (*p* = 0.03), education level (*p* = 0.001), and occupations (*p* < 0.001) between the two groups.

### 3.2. Elevated Blood Lead Levels and Their Association with T2D

**Figure 1:**
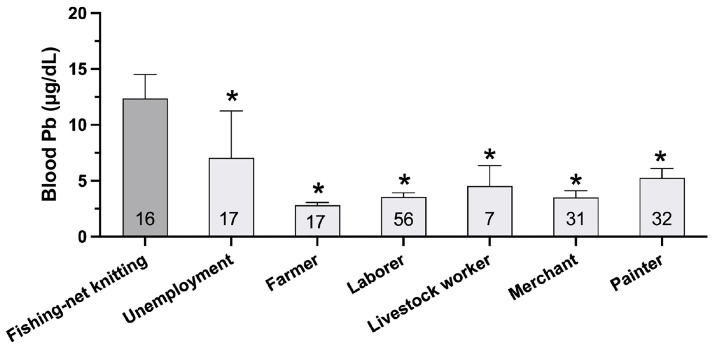
Blood lead levels (BLLs) among different occupational groups. Fishing-net knitters showed the highest mean BLL (12.35 ± 2.15 µg/dL), which was approximately 1.75 times higher than the unemployed individuals (7.04 ± 4.20 µg/dL) and 2.3–4.4 times higher than other occupations including farmers, laborers, livestock workers, merchants, and painters. Number within the bars indicates sample size for each group. Statistical significance was determined using Kruskal–Wallis test, with post hoc comparisons (* *p* < 0.05 compared to Fishing-net knitting group).

Since occupational status showed significant differences between the two groups (Table 1), we hypothesized that blood lead levels would also differ among various occupational groups. Figure 1 demonstrates significant differences in blood lead levels (BLLs) across occupational categories. Fishing-net knitters exhibited the highest mean BLL at 12.35 ± 2.15 µg/dL, which was notably higher than all other occupational groups. This level was approximately 1.75 times higher than that of unemployed individuals (7.04 ± 4.20 µg/dL) and 2.3–4.4 times higher than other occupational categories. Additionally, we hypothesized that exposure to toxic metals, such as lead (Pb) and cadmium (Cd), might cause T2D development. The mean differences in heavy metal concentration in the blood between T2D and non-T2D controls were determined. The results showed that the mean blood lead levels (BLLs) in the T2D and control groups were 5.74 ± 5.64 µg/dL and 3.60 ± 3.71 µg/dL, respectively, indicating that BLLs of T2D were significantly higher than those of the non-diabetic control group (Table 2). However, there was no significant difference in blood cadmium levels between the T2D (0.53 ± 0.6 µg/L) and non-T2D (0.65 ± 0.85 µg/L) groups. The results suggest that BLLs and abdominal fat may contribute to T2D in this study.

### 3.3. Biochemical Characteristics and Body Composition Differences in T2D Patients

The study also evaluated the biochemical characteristics and body composition of the T2D and non-T2D groups, as shown in Table 2. The fasting blood sugar (FBS) levels in the T2D group were 1.77-fold higher than those in the control group. Additionally, the T2D group had an average systolic blood pressure value of 141.3 mmHg, which was higher than that of the non-T2D group (134.9 mmHg). These results suggest that elevated blood sugar levels may contribute to increased systolic blood pressure among T2D patients. Moreover, the body composition, including basal metabolic rate (BMR), percentage of body fat, visceral fat rating scale, bone mass, and total body water, was also determined. Only the percentage of body fat showed a statistically significant difference between the two groups, with a *p*-value of 0.01 (Table 2).

In our study, we observed a correlation between higher blood lead levels (BLLs) and type 2 diabetes (T2D) (Table 2). We compared the differences in socio-demographic characteristics and influencing factors between groups with BLLs > 3 µg/dL and those with BLLs < 3 µg/dL (Table 3). Significant disparities in education (*p* < 0.001), monthly income (*p* = 0.005), and occupations (*p* < 0.001) were found between T2D and non-T2D groups. Specifically, we discovered that compared to unemployment, color painters and fishing net knitters were more likely to exhibit higher BLLs, with odds ratios of 6.9 (95% CI, 1.85–25.76, *p* = 0.004) and 16.8 (95% CI, 2.74–102.87, *p* = 0.002), respectively.

**Figure 2:**
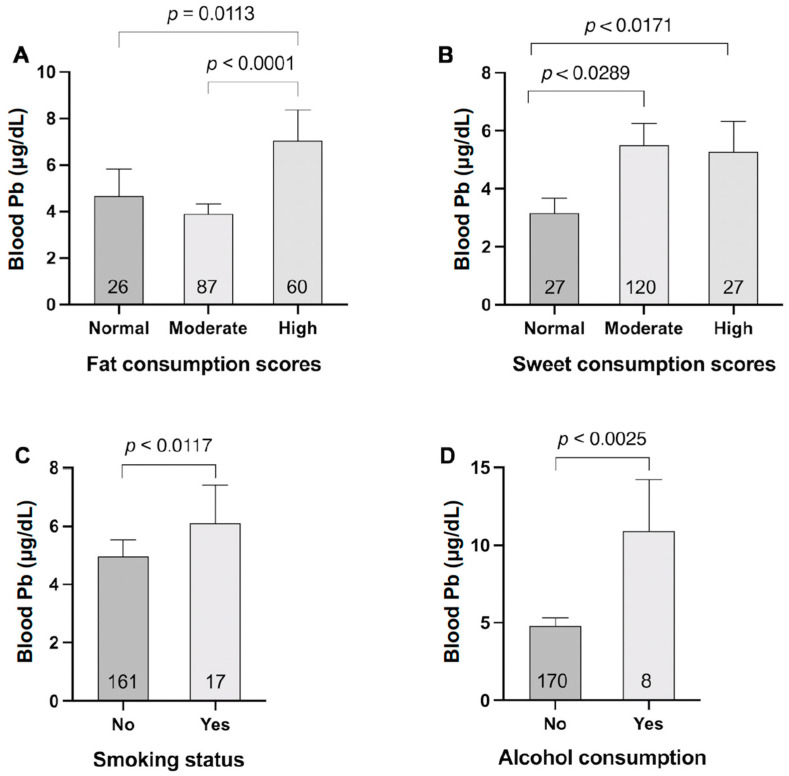
Blood lead levels (BLLs) across dietary and lifestyle factors. (**A**) Mean BLLs by fat consumption categories, showing significantly higher levels in high-fat group compared to normal (*p* = 0.0113) and moderate groups (*p* < 0.0001). (**B**) BLLs across sweet consumption groups, with significantly elevated levels in moderate (*p* < 0.0289) and high consumption groups (*p* < 0.0171) compared to normal. (**C**) BLLs by smoking status, revealing significantly higher levels in smokers (*p* < 0.0117). (**D**) BLLs comparing non-drinkers and drinkers, showing significantly elevated levels in drinkers (*p* < 0.0025). Data shown as mean ± SEM. Numbers within bars indicate sample size for each group.

This suggests that tobacco use and the consumption of sugar- and fat-rich foods may elevate BLLs in individuals. Compared to non-consumption, smoking, and alcohol consumption were associated with higher odds of having BLLs ≥ 3 µg/dL, with odds ratios of 4.17 (95% CI, 1.31–13.37, *p* = 0.016) and 8.56 (95% CI, 1.03–71.16, *p* = 0.047), respectively (Table 4). Figure 2 shows that mean BLLs among smokers (6.11 ± 1.30 µg/dL) and alcohol consumers (10.92 ± 3.34 µg/dL) were 1.12 and 2 times higher, respectively, compared to non-consumers.

High-fat consumption scores were also more likely to correspond with BLLs ≥ 3 µg/dL (OR = 4.4, 95% CI, 1.66–11.73, *p* = 0.003) compared to normal and medium fat consumption scores. Participants with moderate and high sugar consumption scores demonstrated a higher proportion of BLLs ≥ 3 µg/dL compared to those with normal (OR = 2.85, 95% CI, 1.13–7.26, *p* = 0.027) and high (OR = 4.15, 95% CI, 1.31–13.17, *p* = 0.015) sugar consumption scores.

In addition to fishing net knitting, the food, and beverages consumed by participants may be contaminated with lead (Pb). Although blood chromium (Cr) levels in our study did not show a significant association with T2D, blood Cr levels were found to be associated with BLLs ≥ 3 µg/dL, with an odds ratio of 2.7 (95% CI, 1.42–5.23, *p* = 0.003). After adjusting for socio-demographic characteristics (gender, age, marital status, monthly income, BMI, and BMR), our study revealed that co-exposure to lead (Pb) and cadmium (Cd) contributed to the development of T2D. This finding underscores the importance of considering multiple factors, such as occupation, lifestyle, and dietary habits, when assessing the relationship between environmental exposures and health outcomes like T2D. Further research is needed to better understand the underlying mechanisms and to develop effective interventions for reducing BLLs and the risk of T2D in susceptible populations.

## 4. Discussion

This case–control study investigated the relationship between socio-demographic factors, body composition, and environmental heavy metal exposure with type 2 diabetes (T2D) risk among individuals working in lead-related occupations and residing near fishing communities in Nakhon Si Thammarat province.

The study found that education, occupation, and marital status were associated with T2D risk, with occupational classifications significantly influencing prevalence rates. Consistent with prior research, professions involving reduced physical activity, such as professional driving, manufacturing, and labor-intensive work, demonstrated a higher T2D risk [21,22,23,24]. Among occupational groups, fishing net knitters exhibited the highest BLLs (12.35 ± 2.15 µg/dL), followed by color painters (5.25 ± 0.84 µg/dL). These findings suggest that certain professions pose a significant risk for lead exposure, potentially increasing T2D risk. Similar trends were observed in laborers and painters within their initial employment months [22]. Factors such as gender (*p* < 0.021), education (*p* < 0.001), and monthly income (*p* = 0.005) influenced Pb exposure, supporting findings by Kim JH et al. (2017) [25]. Additionally, marital status independently correlated with T2D incidence, aligning with findings from de Oliveira et al. (2020) [26].

Higher fat mass, particularly abdominal fat was significantly associated with T2D risk (Table 1), reinforcing the established link between obesity and diabetes [27,28]. Obesity-induced inflammation alters adipose tissue function, contributing to insulin resistance [29].

Elevated blood lead levels (BLLs) were significantly higher among individuals with T2D (Table 2), supporting previous studies that identified lead exposure as a diabetes risk factor [11,12,30,31]. Prolonged exposure to heavy metals such as lead (Pb) and cadmium (Cd) remains an environmental concern [32], with studies linking elevated Pb and Cd levels to T2D [33,34]. Furthermore, individuals with high Cd levels were more likely to have increased Pb levels (OR: 4.8, 95% CI, 1.25–18.4, *p* = 0.022), in agreement with Yimthiang et al. (2022) [35].

Smoking and alcohol consumption were linked to elevated BLLs (Table 3 and Table 4, Figure 2), possibly due to Pb contamination in tobacco products [2,36]. Moreover, a high intake of sweets and fats was significantly associated with BLLs > 3 µg/dL (ORs: 4.15 and 4.45, respectively), indicating potential dietary Pb exposure [37,38]. Certain sweets and seafood products have been reported to contain lead contamination, potentially explaining this association [37,38]. These findings highlight the importance of dietary habits in heavy metal exposure and metabolic health.

Participants with BLLs > 3 µg/dL had 28% higher fasting blood sugar (FBS) levels compared to those who had BLLs < 3 µg/dL (148.6 vs. 115.9 µg/dL). These results suggested Pb exposure may impair glycemic control. Lead may contribute to hyperglycemia through increased gluconeogenesis and insulin resistance [3,39]. However, no significant differences in blood pressure were noted between high and low BLL groups, differing from findings by Lv Y et al. (2018) [39]. This suggests that the exposure levels in our cohort may not have reached a toxic threshold. Given that Pb exposure affects glucose metabolism, routine BLL monitoring is recommended for T2D risk assessment and prevention [39,40,41,42].

### 4.1. Cd Exposure and T2D Risk

We have observed elevated blood lead (Pb) levels in only diabetic patients, while blood cadmium (Cd) levels were similar in both diabetics and controls. This observation does not rule out the potential role of Cd exposure because blood Cd reflects more recent exposure. Indeed an association of long-term Cd exposure, measured as urinary Cd excretion rate, and an enhanced risk of T2D has been observed in studies from the U.S. [43,44,45]. After adjustment for potential confounders, smoking included, urinary Cd excretion rates of 1–2 μg/g creatinine were associated with a 1.48-fold and 1.24-fold increase in the risk of having prediabetes, defined as fasting plasma glucose ≥ 110 mg/dL, and diabetes, respectively [43]. The risk of having prediabetes rose 3.4-fold in men with obesity and urinary Cd excretion rate in quartile 4, compared to those with a normal weight and having urinary Cd excretion rate in the bottom quartile [44]. These results suggest interactions between Cd exposure and adiposity in men. In another study on the representative of the U.S. population, an increased risk of prediabetes was associated with urinary Cd levels ≥ 0.7 µg/g creatinine after adjustment for covariates [45]. Furthermore, finding from the Dutch prospective cohort of 226 patients with T2D supported the premise that Cd exposure promoted progressive kidney function deterioration to end-stage kidney disease, when dialysis or a kidney transplant is essential for survival [46].

### 4.2. Perspectives for Clinical Practice

This study highlights the importance of environmental and occupational exposure in T2D development. Routine BLL screening, particularly for high-risk occupations, could enhance diabetes management [47]. Lead exposure impairs insulin function and may influence the efficacy of hypoglycemic agents [48]. Healthcare professionals should integrate environmental risk assessments into diabetes management, employing multidisciplinary approaches involving endocrinologists, occupational health specialists, and dietitians. Workplace safety policies and public health initiatives should address lead exposure risks, promoting stricter environmental monitoring and occupational safety standards [48].

### 4.3. Limitations

This study is limited by its regional focus on Pakpoon district, Thailand, restricting generalizability. Selection bias may have arisen due to participant exclusions for incomplete data. The small and gender-imbalanced sample may also limit robustness. Given Pb’s short half-life, a single BLL measurement may not reflect long-term exposure [49,50]. Lead is primarily stored in bone and can be mobilized into the bloodstream based on physiological and environmental factors [49,50]. More comprehensive biomarkers, such as bone lead measurements, are needed to assess chronic exposure. Additionally, nutritional deficiencies can also influence Pb absorption and retention, complicating its relationship with T2D risk [50]. Furthermore, the cross-sectional design prevents causal inference. Future longitudinal studies are needed to assess chronic lead exposure effects and its interplay with other risk factors. Unaccounted confounders, such as dietary habits and genetic predispositions, may also influence results. Despite these limitations, our findings underscore key associations that warrant further research using more robust methodologies and larger sample sizes.

## 5. Conclusions

Exposure to lead and cadmium may potentially contribute to the increasing incidence of T2D, with lead exposure significantly associated with traditional socio-demographic risk factors and consumption behavior factors. Although overall lead exposure levels have decreased in recent years, persistent lead exposure continues to occur in low socioeconomic populations. Further research is needed to establish the relationship between food preferences and lead exposure, the impact of lead and cadmium co-exposure on metabolic health, and the underlying mechanisms involved.

## Figures and Tables

**Table 1:** Demographic characteristics, socioeconomic status, and dietary consumption behaviors of participants with type 2 diabetes and non-diabetic controls.

Variables	Type 2 Diabetes		Non-Diabetic Control		*p*-Value
	*n* = 88		*n* = 90		
	Mean or *n*	SD or % ^b^	Mean or *n*	SD or % ^b^	
Women	71	80.7	73	81.1	0.92
Age, years	60	9	59	10	0.55
BMI, kg/m^2^	26.06	4.9	24.74	4.3	0.058
Waist circumference, cm	95.15	13.1	87.57	10.1	<0.001 *
Marital status					
Married	50	56.8	65	72.2	0.03 *
Education					
Primary school	74	84.1	54	60.0	0.001
Secondary school	12	13.6	24	26.7	
College and university	2	2.3	12	13.3	
Occupations					
Unemployment	3	3.4	14	15.6	<0.001 *
Color painter	28	31.8	3	3.3	
Merchant	17	19.3	14	15.6	
Livestock worker	3	3.4	4	4.4	
Laborer	25	28.4	31	34.4	
Fishing-net knitting	7	8.0	10	11.1	
Farmer	5	5.7	12	13.3	
Fishery	0	0.0	2	2.2	
Income (Baht/month)					
<9000	70	79.5	63	70.0	0.142
≥9000	18	20.5	27	30.0	
Smoking	7	8.0	10	11.1	0.474
Alcohol consumption	6	6.8	2	2.2	0.141
Sweet consumption score ^a^					
Normal	13	14.8	14	16.1	0.832
Moderate	60	68.2	61	56.3	
High	15	17.0	12	27.6	
Fat consumption score ^a^					
Normal	12	13.8	14	16.1	0.113
Moderate	38	43.7	49	56.3	
High	15	17.0	12	27.6	
Fat consumption score ^a^					
Normal	12	13.8	14	16.1	0.113
Moderate	38	43.7	49	56.3	
High	37	42.5	24	27.6	
Salt consumption score ^a^					
Normal	7	8.0	9	10.3	0.401
Moderate	76	87.4	70	80.5	
High	4	4.6	8	9.2	

BMI = Body mass index; ^a^ Sweet, fat, and salt consumption scores were obtained by questionnaires, and they were considered normal, moderate, and high using the Thai RDA recommendation [20]. Data for blood lead level (Pb) are geometric means ± SD. Data for all other continuous variables are arithmetic means ± SD. ^b^ Differences in mean and percentages were assessed with a *t*-test and Chi-square test, respectively. The * *p*-values < 0.05 identifies statistically significant levels.

**Table 2:** Biomedical characteristics and blood concentrations of toxic metals in type 2 diabetes and non-diabetic controls.

Variables	Type 2 Diabetes		Non-Diabetic Control		*p*-Value
	*n* = 88		*n* = 90		
	Mean or *n*	SD or % ^a^	Mean or *n*	SD or % ^a^	
Fasting blood glucose, mg/dL	168.7	67.58	94.87	12.40	<0.001 *
≥110 mg/dL	78	88.6	8	8.9	<0.001 *
SBP, mmHg	141.3	17.07	134.9	16.79	0.006 *
>140	47	53.4	31	35.2	0.025
DBS, mmHg	83.44	9.78	84.09	9.107	0.325
>90	21	23.9	23	26.4	0.391
Toxic metal exposure					
Blood Pb (µg/dL)	5.74	5.64	3.60	3.71	0.001 *
≥3.0 µg/dL	25	28.1	58	65.9	<0.001 *
Blood Cd (µg/L)	0.53	0.60	0.65	0.85	0.158
≥0.4 µg/L	56	62.2	60	68.2	0.404 *
Basal metabolic rate (BMR) ^b^					
Low	43	48.9	31	37.8	0.34
Normal	31	35.2	36	43.9	
High	14	15.9	15	18.3	
% Body fat ^c^					
Low	11	12.5	30	33.3	0.01 *
Normal	38	43.2	39	43.3	
High	21	23.9	16	17.8	
Very high	18	20.5	5	5.6	
Visceral Fat Rating Scale ^d^					
Normal	53	60.2	62	68.9	0.46
High	29	33.0	24	26.7	
Very high	6	6.8	4	4.4	
Bone Mass ^e^					
Low	27	23.5	28	23.7	0.951
Normal	88	76.5	90	76.3	
Water ^f^					
Normal	70	79.5	75	83.3	0.52
Low	18	20.5	15	16.7	

^a^ Differences in mean and percentages were assessed with *t*-test and Chi-square test, respectively. SBP = Systolic blood pressure; DBP = Diastolic blood pressure; ^b^ BMR; <1176–1386 kcal, low; 1597–1808 kcal, normal; and 1809–2123 kcal, high for male; and 1057–1192 kcal, low; 1330–1467 kcal, normal; and 1468–1671 kcal, high for female; ^c^ % Body fat: low < 20%, normal: 20–35%, high: 35–40%, very high: >40%; ^d^ Visceral rating scale, normal (1–9), high (10–14), and very high >15; ^e^ Average bone mass female, <45 kg = 1.8, 45–60 kg = 2.2 kg, and >60 kg = 2.5 kg were grouped as normal; Average bone mass male, <60 kg = 2.5 kg, 60–75 kg, 2.9, and >75 kg = 3.2 kg were grouped as normal; ^f^ Healthy total body water percentage, female: 45–60% and male: 50–65% were considered as normal. The * *p*-values < 0.05 identify statistically significant levels.

**Table 3:** Comparing socio-demographic characteristics, environmental exposure, and biomedical parameters of participants stratified by their blood lead levels.

	Blood Lead Levels				*p*-Value
Variables	≥3 µg/dL, *n* = 83		<3.0 µg/dL, *n* = 94		
	Mean or *n*	SD or % ^b^	Mean or *n*	SD or % ^b^	
Women	61	73.5	82	87.2	0.021 *
SBP, mmHg	138.7	17.32	137.8	17.00	0.366
DBP, mmHg	83.85	9.268	83.74	9.594	0.899
MAP, mmHg	101.3	11.34	101.5	10.70	0.425
Blood Cd	0.6956	0.7431	0.4997	0.7247	0.006 *
≥0.4 µg/L	64	71.1	52	55.3	0.002 *
Fasting blood glucose (mg/dL)	148.6	70.31	115.9	47.18	<0.001 *
≥110	51	61.4	35	37.2	0.001 *
Education					
Primary school	72	86.8	55	58.5	<0.001 *
Secondary school	9	10.8	27	28.3	
University	2	2.4	12	12.8	
Occupations					
Unemployment	5	6.0	12	12.8	<0.001*
Painter	23	27.7	8	8.5	
Merchant	9	10.8	22	23.4	
Livestock worker	2	2.4	5	5.3	
Laborer	22	26.5	34	36.2	
Fishing-net knitting	14	16.9	2	2.1	
Farmer	6	7.2	11	11.7	
Fishery	2	2.4	0	0.0	
Monthly income (Baht)					
<9000	70	84.3	62	66.0	0.005 *
≥9000	13	15.7	32	34.0	
Smoking	13	15.7	4	4.3	0.010 *
Alcohol consumption	7	8.4	1	1.1	0.019 *
Sweet consumption ^a^					
Normal	7	8.4	20	22.0	0.033 *
Moderate	60	72.3	60	65.9	
High	16	19.3	11	12.1	
Fat Score ^a^					
Normal	9	11.0	17	18.7	<0.001 *
Moderate	31	37.8	56	61.5	
High	42	51.2	18	19.8	

^a^ Sweet, fat, and salt consumption scores were determined by related diet-related habits questionnaire on liking for sweet, fatty foods and seasoning with salt. The scores were devised into normal, modulated, and high based on Thai RDA recommendations [20]. ^b^ Differences in mean and percentages were assessed with a *t*-test and Chi-square test, respectively. The * *p*-values < 0.05 identifies statistically significant levels.

**Table 4:** Socio-economic, behavioral, and environmental risk factors for high blood lead concentrations.

Variables	Crude Odds for Blood Pb ≥ 3 µg/dL					^a^ Adjusted Odds for Blood Pb ≥ 3 µg/dL				
	β Coefficients (SE)	ORs	95% CI		*p*-Valve	β Coefficients (SE)	ORs	95% CI		*p*-Valve
			Lower	Upper				Lower	Upper	
Blood cadmium	1.001 (0.334)	2.72	1.42	5.23	0.003 *	1.569 (0.685)	4.80	1.25	18.40	0.022 *
Occupations										
Unemployment	Ref					Ref				
Color painter	1.932 (0.672)	6.90	1.85	25.76	0.004 *	0.472 (1.135)	1.60	0.17	14.82	0.678
Merchant	−0.018 (0.663)	0.98	0.27	3.60	0.978	−0.670 (1.127)	0.51	0.06	4.65	0.552
Livestock worker	−0.041 (0.992)	0.96	0.14	6.70	0.967	1.052 (1.746)	2.86	0.09	87.78	0.547
Laborer	0.440 (0.598)	1.55	0.48	5.02	0.462	0.826 (1.061)	2.28	0.29	18.26	0.436
Fishing-net knitting	2.821 (0.925)	16.80	2.74	102.87	0.002 *	3.159 (1.436)	23.55	1.41	393.14	0.028 *
Farmer	0.269 (0.735)	1.30	0.31	5.53	0.714	2.424 (1.394)	11.30	0.74	173.58	0.082
Education										
Primary school	Ref					Ref				
Secondary school	−1.368 (0.425)	0.25	0.11	0.59	0.001 *	−2.873 (1.013)	0.06	0.01	0.41	0.005 *
University	−2.061 (0.784)	0.12	0.03	0.59	0.009 *	−2.260 (1.424)	0.10	0.01	1.69	0.112
Behavior consumption										
Smoking	1.430 (0.594)	4.17	1.31	13.37	0.016 *	1.938 (1.477)	0.36	0.03	3.93	0.402
Alcohol drinking	2.148 (1.080)	8.56	1.03	71.16	0.047 *	3.825 (2.039)	45.85	0.84	2493.10	0.061
Sweet Score										
Normal	Ref					Ref				
Moderate	1.050 (0.476)	2.85	1.13	7.26	0.027 *	1.227 (0.789)	3.41	0.73	16.00	0.120
High	1.425 (0.588)	4.15	1.31	13.17	0.015 *	1.792 (1.028)	6.00	0.80	45.05	0.081
Fat Score										
Normal	Ref					Ref				
Moderate	0.045 (0.469)	1.04	0.42	2.62	0.924	0.022 (0.750)	1.02	0.24	4.44	0.976
High	1.483 (0.499)	4.40	1.66	11.73	0.003 *	1.633 (0.896)	5.12	0.88	29.67	0.068

Odds ratios (ORs) for high blood were determined by binary logistic regression analysis, and * *p*-value < 0.05 was identified as a significant risk associated with high blood Pb ≥ 3 µg/dL ^a^ Model adjusted for socio-demographic characteristics (gender, age, marital status, monthly income, BMI, and BMR).

## Data Availability

All data are contained within this article. The data are not publicly available due to confidentiality and ethical considerations but can be provided upon reasonable request.

## Supplementary Materials

The following supporting information can be downloaded at: https://www.mdpi.com/article/10.3390/nu17040710/s1, Table S1: STROBE statement checklist of items included in the observational studies.
