# Supplementary material for: Concurrent Lead and Cadmium Exposure Among Diabetics: A Case-Control Study of Socio-Demographic and Consumption Behaviors
Source: Nutrients. 2025 Feb 17;17(4):710. doi: 10.3390/nu17040710 (PMC11858647; doi:10.3390/nu17040710)
Supplement: Supplementary file 1 [file nutrients-17-00710-s001.zip › nutrients-3474369-supplementary.pdf]

Supplementary Table S1: STROBE Statement—a checklist of items included in the observational studies

	Item No.		Recommendation	Page No.	Relevant text from the manuscript
**Title:** Concurrent Lead and Cadmium Exposure among Diabetics: A Case-Control Study of Socio-Demographic and Consumption Behaviors	1	*(a)*	**Indicate the study's design with a commonly used term in the title or the abstract** **Case-control study** explores the relationship between exposure to lead (Pb) and cadmium (Cd) and the heightened risk of T2D in areas with heavy metal contamination.	1	
		(b)	**Provide in the abstract an informative and balanced summary of what was done and what was found.** This study highlights the connection between lead exposure, socio-demographic factors, and consumption habits, which may contribute to T2D incidence prediction. These results underscore the need for diabetes care strategies that incorporate environmental and occupational exposure risk assessments.	1	
**Introduction:** The global prevalence of diabetes is rising, making it a major public health concern. The World Health Organization estimates that 250 million people have diabetes, with projections surpassing 360 million by 2030. This growing burden has significant socioeconomic and healthcare implications. Type 2 diabetes (T2D) affects multiple organs, leading to complications such as stroke, heart attacks, and kidney failure. Established risk factors include genetics, obesity, physical inactivity, smoking, and alcohol use. However, emerging evidence suggests that environmental exposure to heavy metals may also contribute to T2D risk. Heavy metals pose health hazards due to their toxicity and bioaccumulation. They originate from geogenic, industrial, agricultural, and domestic sources. Human exposure occurs through contaminated food, water, occupation, and residence in high-risk areas. Research links lead, cadmium, mercury, and arsenic exposure to chronic diseases, including T2D and hypertension. Epidemiological studies suggest an association between urinary cadmium levels and T2D, but findings remain inconsistent due to study design differences. Thailand has a high diabetes prevalence (9.9%), with 30,000 annual deaths. Lead contamination is documented in high-risk communities. This study aims to explore the association between lead and cadmium exposure and T2D, identifying risk factors to improve diabetes management and prevention.				2	
Background/rationale	2		The global prevalence of diabetes is rising, with projections surpassing 360 million cases by 2030. This increasing burden poses significant healthcare and socioeconomic challenges. Type 2 diabetes (T2D) affects multiple organs, leading to complications such as stroke, heart attacks, and kidney failure. While genetic and lifestyle factors are established risks, emerging evidence suggests that environmental exposure to heavy metals may contribute to T2D development.	2	

		Heavy metals, including lead and cadmium, are widely distributed through industrial and agricultural sources. Given Thailand's high diabetes prevalence and documented lead contamination, this study investigates the link between heavy metal exposure and T2D risk.	
Objectives	3	This study aims to investigate the association between lead and cadmium exposure and type 2 diabetes (T2D) through a case-control design. Specific objectives include assessing blood lead and cadmium levels in individuals with and without T2D and identifying key risk factors. We hypothesize that higher exposure to these heavy metals is associated with an increased risk of T2D.	2
**Methods**			
Study design	4	This case-control study included 88 diabetic and 90 non-diabetic participants from a high-contamination region. Blood and urine samples were analyzed for lead and cadmium levels, with sociodemographic and lifestyle data collected through structured interviews. Statistical analysis, including logistic regression, was performed to assess associations between heavy metal exposure and T2D risk, adjusting for confounding factors.	3-4
Setting	5	This study was conducted in Thailand, focusing on regions with high lead and cadmium contamination, particularly Nakhon Si Thammarat province. Participant recruitment and data collection took place from January to December 2020. The study assessed environmental heavy metal exposure and its association with type 2 diabetes through a structured epidemiological approach.	3-4
Participants	6	(a) This case-control study included 88 diabetic cases and 90 non-diabetic controls, matched by age and gender. Cases were selected based on diagnosed type 2 diabetes, while controls were recruited from local health canter. The study focused on populations in high heavy metal contamination areas to assess exposure-related diabetes risk factors.	3-4
		(b) This case-control study matched cases and controls by age and gender, with a 1:1 ratio. A total of 88 individuals diagnosed with type 2 diabetes were matched with 90 non-diabetic controls from the same community to ensure comparability in assessing the association between heavy metal exposure and diabetes risk.	3-4
Variables	7	The primary outcome is type 2 diabetes, diagnosed based on fasting plasma glucose ≥126 mg/dL or HbA1c ≥6.5%. Exposures include blood lead and cadmium levels. Predictors include	3-4

		occupational and environmental exposure. Potential confounders, such as obesity and smoking, were controlled. Effect modifiers include genetic predisposition and lifestyle factors.	
Data sources/ measurement	8*	Data on diabetes status were obtained through fasting plasma glucose and HbA1c tests. Blood lead and cadmium levels were measured using atomic absorption spectrometry. Demographic, lifestyle, and occupational exposure data were collected via structured questionnaires. Standardized laboratory methods ensured comparability across study groups.	3-4
Bias	9	Selection bias was minimized through a matched case-control design based on age, sex, and residency. Recall bias was addressed by using biomarker-based exposure assessment rather than self-reports. Confounding factors, including lifestyle and occupational exposures, were controlled through multivariate statistical analysis. Standardized protocols ensured consistency in data collection and laboratory measurements.	3-4
Study size	10	The study size was determined using power calculations to detect a significant association between heavy metal exposure and T2D risk. Sample size estimation considered expected effect size, prevalence of exposure, and statistical power of 80% with a 95% confidence level. Matching cases and controls ensured adequate comparability while maintaining sufficient statistical precision.	3-4

Continued on next page

Quantitative variables	11	Quantitative variables, such as blood lead and cadmium concentrations, were analyzed as continuous variables and categorized into quartiles for subgroup comparisons. Mean and standard deviation were used for descriptive statistics, while logistic regression models assessed associations with T2D. Groupings were chosen based on established exposure thresholds and prior epidemiological evidence.	3-4
Statistical methods	12	(*a*) Statistical methods include logistic regression to assess associations between heavy metal exposure and T2D, adjusting for confounders such as age, sex, obesity, and lifestyle factors. Multivariate models were used to control for potential confounders. Stratification by metal exposure levels and adjustment for co-exposure were applied to minimize bias in estimating the relationship between exposure and diabetes risk.	3-4
		(*b*) Subgroup analyses were conducted to examine potential differences in the relationship between heavy metal exposure and T2D across age, sex, and occupation. Interaction effects were assessed to determine if the association between exposure and diabetes risk varied by co-exposure to other metals, adjusting for these variables to better understand their combined influence on diabetes prevalence.	3-4
		(*c*) Missing data were handled through multiple imputation methods, ensuring that any incomplete information did not bias the results. This approach allowed for the estimation of missing values based on observed data patterns, helping maintain the robustness of the study and enhancing the accuracy of the associations between heavy metal exposure and type 2 diabetes risk	3-4
		(*d*) In this case-control study, matching of cases and controls was performed based on key demographic and health characteristics, such as age, gender, and other relevant risk factors. This approach minimized confounding and ensured that comparisons between groups were valid, allowing for more accurate conclusions about the relationship between heavy metal exposure and type 2 diabetes risk.	3-4
		(*e*) Sensitivity analyses were conducted to assess the robustness of the findings, particularly in relation to potential biases or confounders. These analyses involved testing the impact of different assumptions, such as varying exposure levels or adjusting for additional covariates, to determine the stability of the observed associations between heavy metal exposure and type 2 diabetes risk.	3-4
**Results**			
Participants	13*	**(a) Report the number of individuals at each stage of the study**	4-11

			The study enrolled 178 participants, including 88 T2D cases and 90 controls. Blood lead levels were significantly higher in T2D participants (5.74 ± 5.64 µg/dL) compared to controls (3.60 ± 3.71 µg/dL). Differences in sociodemographic factors like education, occupation, and smoking status were observed between the groups, with significant variations in blood lead levels.	
		**(b)**	**Give reasons for non-participation at each stage**<br>Non-participation at each stage can be attributed to various factors, such as time constraints, health issues, lack of interest, or logistical challenges. In particular, the study saw higher non-participation rates among certain occupational groups, as well as those with lower education and income levels, which could have impacted their ability or willingness to engage.	4-11
		**(c)**	**Consider the use of a flow diagram**<br>To enhance the clarity of the results, the graphical abstract flow diagram was used to illustrate the relationship between blood lead levels (BLLs), socio-demographic characteristics, and their association with T2D. This diagram highlights significant differences in occupation, education, and consumption behaviors (e.g., smoking, fat intake), emphasizing their impact on BLLs and T2D risk.	4-11
Descriptive data	14*	**(a)**	**Give characteristics of study participants (eg demographic, clinical, social) and information on exposures and potential confounders**<br>The study included 178 participants (88 T2D cases and 90 controls), predominantly women (81%). Participants had an average age of 59-60 years. Key differences between T2D and control groups were noted in waist circumference, marital status, education, and occupation. Blood lead levels were significantly higher in the T2D group, with occupational exposure playing a key role in elevated lead levels.	4-11
		**(b)**	**Indicate the number of participants with missing data for each variable of interest**<br>In the study, there were 25 participants (28.1%) in the T2D group and 58 participants (65.9%) in the control group with blood lead levels (BLLs) ≥ 3.0 µg/dL. No other specific missing data points were mentioned in the results.	4-11
		**(c)**	***Cohort study***<br>The study had a follow-up period of unspecified length, focusing on participants' blood lead levels, socio-demographic characteristics, and T2D status. The study included 178	4-11

		participants (88 T2D cases, 90 controls) and compared various health and exposure metrics across these groups, particularly highlighting significant differences in blood lead levels, BMI, and dietary habits.	
Outcome data	15*	***Cohort study***<br>T2D participants had significantly higher waist circumference (95.15 cm vs. 87.57 cm, p < 0.001) and blood lead levels (5.74 ± 5.64 µg/dL vs. 3.60 ± 3.71 µg/dL, p = 0.001) compared to controls. Fasting blood glucose was 1.77 times higher in T2D patients, with a significant association between elevated BLLs, education, and occupational exposure.	4-11
		Case-control study<br>The case-control study enrolled 178 participants (88 T2D cases, 90 controls) matched by age and gender. The T2D group had a significantly higher waist circumference (95.15 cm vs. 87.57 cm, p < 0.001). Significant differences were observed in marital status (p = 0.03), education (p = 0.001), and occupations (p < 0.001), but not in BMI, income, smoking, or dietary habits.	4-11
		Cross-sectional study—Report numbers of outcome events or summary measures	
Main results	16	***(a)*** **Give unadjusted estimates and, if applicable, confounder-adjusted estimates and their precision (eg, 95% confidence interval). Make clear which confounders were adjusted for and why they were included**<br>The mean waist circumference was significantly higher in T2D cases (95.15 cm) than in controls (87.57 cm, p < 0.001). Blood lead levels were also higher in T2D cases (5.74 ± 5.64 µg/dL) than in controls (3.60 ± 3.71 µg/dL, p = 0.001). Odds ratios indicated increased BLLs in color painters (OR = 6.9, 95% CI: 1.85-25.76) and fish-net knitters (OR = 16.8, 95% CI: 2.74-102.87).	4-11
		(b) Report category boundaries when continuous variables were categorized<br>The study categorized continuous variables such as BMI, waist circumference, and biochemical parameters into specific ranges, reporting category boundaries. For example, body fat percentage was classified as low (<20%), normal (20-35%), high (35-40%), and very high (>40%), while fasting blood glucose was categorized based on a threshold of 110 mg/dL.	4-11
		(c) **If relevant, consider translating estimates of relative risk into absolute risk for a meaningful period**	4-11

		The study found that individuals with T2D had significantly higher mean blood lead levels (BLLs) than controls (5.74 µg/dL vs. 3.60 µg/dL, p=0.001). If relevant, this relative difference could be translated into an absolute risk estimate over a meaningful period, helping to contextualize the health impact of elevated BLLs on T2D development.	
Other analyses	17	The study conducted subgroup and sensitivity analyses to explore associations between blood lead levels (BLLs), socio-demographics, and type 2 diabetes (T2D). Significant differences in BLLs were observed across occupational categories, with fish-net knitters exhibiting the highest levels. Additionally, elevated BLLs were linked to higher fasting glucose, smoking, alcohol consumption, and dietary patterns, indicating potential environmental risk factors for T2D.	4-11
**Discussion**			
Key results	18	The study identified significant associations between socio-demographic factors, occupational classifications, and type 2 diabetes (T2D) risk. Increased percent fat mass and elevated blood lead levels (BLLs) were strongly correlated with T2D. Occupational exposure, particularly in fishing net knitting, contributed to high BLLs. Smoking, alcohol consumption, and dietary habits influenced lead exposure, highlighting environmental and lifestyle factors in T2D development.	12-14
Limitations	19	The study has several limitations that may introduce bias or imprecision. The restricted geographic scope limits generalizability and selection bias may arise from the exclusion of certain participants. The small sample size, particularly the low proportion of male participants, may skew results. Additionally, the cross-sectional design prevents causal inference, and unmeasured confounders, such as genetic factors, may influence findings.	12-14
Interpretation	20	This study highlights potential associations between occupational lead exposure, body composition, and type 2 diabetes (T2D) risk. While findings align with previous research, limitations include sample size, regional constraints, and gender imbalances. These factors warrant caution in generalizing results. Further longitudinal studies and larger, more diverse samples are needed to confirm causality and explore underlying mechanisms.	12-14
Generalisability	21	The generalisability of the study results may be limited due to its regional focus on Pakpoon district, Thailand, and a small, potentially biased sample size, especially with the underrepresentation of male participants. This affects external validity, suggesting that further studies in diverse regions and larger, more balanced samples are necessary to confirm the findings.	12-14
**Other information**			

Funding	22	The study was funded by the Research Grant WU-IRG-63-026 from Walailak University, Thailand. Additionally, Jonah Bawa Adokwe received a Ph.D. Excellence Scholarship (Contract No. PE05/2021) from Walailak University. The funders were involved in funding acquisition but had no role in study design, data collection, or analysis.	15

*Give information separately for cases and controls in case-control studies and, if applicable, for exposed and unexposed groups in cohort and cross-sectional studies.

Note: An Explanation and Elaboration article discusses each checklist item and gives methodological background and published examples of transparent reporting. The STROBE checklist is best used in conjunction with this article (freely available on the Web sites of PLoS Medicine at http://www.plosmedicine.org/, Annals of Internal Medicine at http://www.annals.org/, and Epidemiology at http://www.epidem.com/). Information on the STROBE Initiative is available at www.strobe-statement.org.
